# Comparing recurrent convolutional neural networks for large scale bird species classification

**DOI:** 10.1038/s41598-021-96446-w

**Published:** 2021-08-24

**Authors:** Gaurav Gupta, Meghana Kshirsagar, Ming Zhong, Shahrzad Gholami, Juan Lavista Ferres

**Affiliations:** 1 Ming Hsieh Department of Electrical and Computer Engineering, University of Southern California, Los Angeles, CA 90089 USA; 2 AI for Good Research Lab, Microsoft, Redmond, WA 98052 USA

**Keywords:** Statistics, Biodiversity, Conservation biology, Animal behaviour, Time series, Signal processing

## Abstract

We present a deep learning approach towards the large-scale prediction and analysis of bird acoustics from 100 different bird species. We use spectrograms constructed on bird audio recordings from the Cornell Bird Challenge (CBC)2020 dataset, which includes recordings of multiple and potentially overlapping bird vocalizations with background noise. Our experiments show that a hybrid modeling approach that involves a Convolutional Neural Network (CNN) for learning the representation for a slice of the spectrogram, and a Recurrent Neural Network (RNN) for the temporal component to combine across time-points leads to the most accurate model on this dataset. We show results on a spectrum of models ranging from stand-alone CNNs to hybrid models of various types obtained by combining CNNs with other CNNs or RNNs of the following types: Long Short-Term Memory (LSTM) networks, Gated Recurrent Units (GRU), and Legendre Memory Units (LMU). The best performing model achieves an average accuracy of 67% over the 100 different bird species, with the highest accuracy of 90% for the bird species, Red crossbill. We further analyze the learned representations visually and find them to be intuitive, where we find that related bird species are clustered close together. We present a novel way to empirically interpret the representations learned by the LMU-based hybrid model which shows how memory channel patterns change over time with the changes seen in the spectrograms.

## Introduction

Recent reports of shrinking bird populations world-wide^1,2^ have emphasized the importance of monitoring wild bird populations and protecting biodiversity. With this increasing need, automated audio recorders enable systematic recordings of environmental sounds and have recently opened new opportunities for ecological research and conservation practices. As many bird species have high vocal activities, bioacoustics has become one of the ideal ways to study them. Passive acoustic monitoring (PAM) of biological sounds can provide long-term and standardized data of the composition and dynamics of animal communities. Many bird species produce clear and consistent sounds, thus making acoustic surveys a reliable method to estimate the abundance, density, and occupancy of species^3,4^. Further, visual monitoring is difficult for many small and elusive birds, for cryptic species^5^, and for species found in ecosystems difficult to reach for ecologists^6^. Acoustic monitoring of birds is also helpful for other conservation activities, such as measuring forest restoration^7^, and studying the impact of wild fires^8^.

With the increasing volume of available audio recordings and the development of machine learning algorithms, autonomous classification of animal sounds has recently attracted a wide range of interests. Before deep learning gained wide-spread popularity, prior work had focused on feature extraction from raw audio recordings, followed by some classification models, such as Hidden Markov Model^9,10^, Random Forest^11^, and Support Vector Machines^12^. While these methods demonstrated the successful use of machine learning approaches, their major limitation has been that most of the features need to be manually identified^13^ by a domain expert in order to make patterns more visible for the learning algorithms to work. In comparison, deep learning algorithms try to learn high-level features from the data in an incremental manner, which eliminates the need for domain expertise and hard core feature extraction efforts^14^. Deep learning networks do not require human intervention, as multiple layers in neural networks places data in a hierarchy of different concepts^14^, which ultimately learns from their own mistakes.

**Figure 1:**
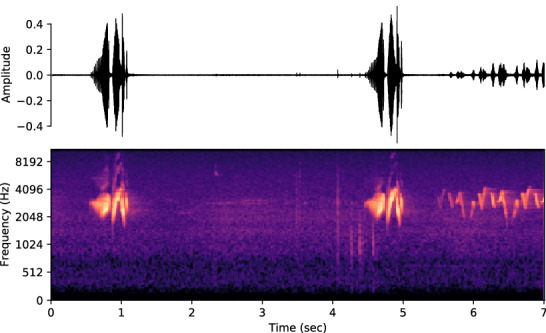
Audio spectrogram representation. The raw audio signal is transformed using the Fourier transform into a mel-spectrogram image. The frequency on the y-axis is in the mel scale.

The use of deep learning for sound detection has spanned multiple domains, ranging from music classification^15,16^ to animal classification/detection (for example, marine species^17,18^, frogs^19^, avian^20,21^, etc.). Among the related call detection and species classification works in the bioacoustics field, most of them adopted the methodology of using Convolutional Neural Networks (CNN) to classify the spectrograms or mel-spectrograms extracted from raw audio clips. These works achieved great success and the deep learning models performed well with high classification accuracy to detect the presence or absence of calls from a particular species, or to classify calls from multiple species. While this method works well by transforming the raw audio into a spectrogram and then treating it as an image classification task, it does not take into consideration the underlying temporal dependence characteristics of the species calls. It is worth noting that, different from the images with real objects, the x- and y-axis of spectrograms have specific implications (i.e., time and frequency, respectively, see Fig. 1), and the time component embedded in the acoustics data shall contain important information for the corresponding classification tasks. Some commonly used data augmentation techniques for image classification, such as rotation and flipping, may not make intuitive sense when applying to spectrograms generated from the acoustics data.

Our work proposes a hybrid deep learning model that incorporates the benefit of convolutional and recurrent neural network models, capturing both spatial and temporal dependence of the bioacoustics data. The contributions of the current work are: (1) our models achieves a better performance than previous ImageNet-based models, and at the same time has 7 times fewer parameters than networks such as VGG16; (2) we present a novel empirical way to interpret the memory channels of the temporal component of our hybrid model; (3) we present a way for ecologists to visualize the learned representations on different bird species.

## Results

### Dataset

For the dataset, we use the bird call classification ‘Cornell Bird Challenge’ (CBC)2020 dataset^22^ along with its extension, which consists of a total of 264 bird species with around 9 to 1778 audio samples per species. For the challenge, CBC2020 obtained the data from https://xeno-canto.org. The raw audio samples vary in length from 5 s to 2 min. Since some classes have very few samples, we chose 100 classes of birds by picking the classes with the highest numbers of samples, and then ensured that each class had at least 100 samples and was close to being balanced. Due to the variable length of the audio samples, we used a fixed-length: the first 7 s of each audio clip, we ignored audios that are shorter, resulting in a total of 15,032 samples across the 100 classes. We settled on the heuristic of taking the first 7 s based on the criterion used for data curation by https://xeno-canto.org which requests bird audio contributors to trim the non-focal sounds and ensure that the specific bird species (focal sound) is heard within the first few seconds of the audio. For training the machine learning models, we split the dataset into 80% training, 10% validation, and 10% test examples. To tackle the over-fitting, a Stratified-KFold resampling technique is used. We performed a 5-fold resampling and the test accuracy results are averaged across these folds. The raw audio clips are transformed to a mel-spectrogram based representation (see Fig. 1 and “Methods”) using the librosa^23^ package.

### Comparing models

We train several variants of hybrid models and compare their average test accuracy using a 5-fold cross-validation to that of the baseline models. Specifically, we compare (i) the ImageNet models VGG16^24^ and ResNets^25^ trained on a single spectrogram of the entire audio clip which we term as ‘stand-alone’ models. Next, (ii) hybrid models with window slides of the raw audio, and then the spectrogram of each slide as an input using convolutional neural network (CNN) for representation and either CNN or recurrent neural network (RNN) for temporal correlation (see “Methods”). In Table 1 we show the test accuracy for stand-alone models as well as hybrid models. For the definitions of CNN and TCNN see section “Methods”. The ImageNet based models (stand-alone) lag behind the hybrid model in test accuracy which shows that explicitly using the temporal component in the models helps bird sound classification. We can make the following conclusions from the results in Table 1: (a) as we increase the complexity of the CNN from CNN1 to CNN3 (going downwards in the table), we see better test accuracy for all the hybrid models; (b) increasing the size of TCNN does not necessarily increase the test accuracy; (c) increasing the size of the hidden state in each RNN (going from 128 to 512) increases the test accuracy for all RNNs; (d) however, increasing the number of layers in the RNN does not necessarily improve the performance. We refer the reader to Supplementary Tables 1, 2 for the complete results. For most of the models, one or two layers results in the best performance across all RNNs. Overall, the temporal block with the Gated Recurrent Unit (GRU) achieves the best accuracy, while using GRU and Legendre Memory Units (LMU) together also gives a similar accuracy to the best model but with less trainable parameters. We discuss the aspect of trainable parameters for each model later in this section.

**Table 1:** Test accuracy comparison on the CBC2020 dataset.

Stand-alone models													
		ResNet18				ResNet50				VGG16			
		0.516				0.537				0.619			
Temporal correlation with CNN/RNN													
Size	TCNN	LSTM			GRU			LMU			GRU+LMU		
		Layers											
		1	2	3	1	2	3	1	2	3	1	2	3
		CNN1											
S	0.49	0.57	0.56	0.55	0.60	0.58	0.58	0.57	0.55	0.54	–	0.57	0.56
L	0.50	0.62	0.61	0.61	0.63	0.64	0.63	0.61	0.61	0.59	–	0.63	0.63
		CNN2											
S	0.56	0.60	0.58	0.55	0.62	0.60	0.60	0.58	0.557	0.56	–	0.59	0.59
L	0.55	0.62	0.61	0.60	0.63	0.63	0.64	0.63	0.62	0.60	–	0.63	0.63
		CNN3											
S	0.58	0.66	0.62	0.58	0.64	0.64	0.62	0.65	0.64	0.63	–	0.63	0.62
L	0.61	0.66	0.63	0.64	0.66	**0.67**	0.65	0.65	0.65	0.64	–	**0.66**	0.65

**Top**: Models without any explicit temporal layer. The input is a single spectrogram from a sound sample. **Bottom**: A comprehensive comparison of models’ test accuracy using CNN/RNN for temporal correlation. The complexity of the CNN used for representation increases from top to bottom. The best accuracy achieved is shown in bold. For each representation CNN*, a small width (S) and a large width (L) temporal layer is shown. For RNNs the S/L refer to the hidden layer size of 128/512, while for TCNN S/L refers to TCNN1/TCNN3.

**Figure 2:**
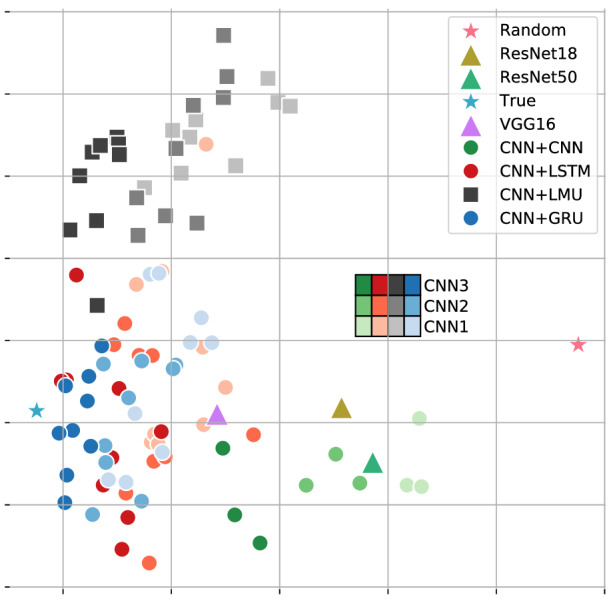
Comparison of models. A PCA plot showing aggregated test outputs ($\in {\mathbb {R}}^{100}$) of various models (see “Comparing models” section for details). The point *True* (green star, left-most along x-axis) denotes the correct $100\times 1$ one-hot encoding test label output, while the *Random* point (right-most along x-axis) denotes the uniform probability (or maximum entropy) $100\times 1$ output. The shades of the points indicate which particular CNN was used in the hybrid model.

In Table 1 we compared the test accuracy of the models, which gives us information about the prediction, i.e the maximum value of the softmax outputs. We then compare the softmax distribution of the models in Fig. 2 in the following manner. First, for each trained model, the softmax outputs of all the test samples are concatenated. Second, the concatenated softmax vectors are then projected along the two dimensions with the maximum variance by performing Principal Component Analysis (PCA). We observe that the hybrid models with CNN for both representation and temporal components are clustered together with the stand-alone models, and are different from the hybrid models that use RNNs for the temporal component. The hybrid models with RNNs that have gating mechanisms like Long Short-Term Memory networks (LSTM) and GRU are very close to each other in the PCA plot. The hybrid models with LMU are clustered together and are away from LSTM and GRU. For reference, we also show the two corner cases of (i) ‘true’, which is the actual one-hot label of the test samples, and (ii) ‘random’ which assigns equal probability to all classes.

**Figure 3:**
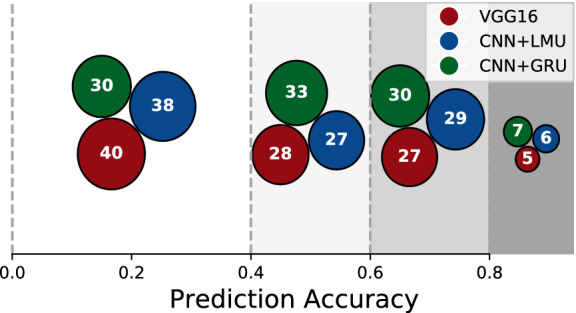
Class-wise predictions. We show, for VGG16, and the best CNN+GRU and CNN+LMU models (from Table 1), the number of classes that each model has the prediction accuracy in the given shaded brackets indicated along the x axis.

For different models, we also show the model complexity in terms of total trainable parameters in Table 2. We conclude that on the CBC2020 dataset, the stand-alone ImageNet-based models with higher trainable parameters do not deliver higher test accuracy. The hybrid models offer dual advantages in terms of less model complexity as well as higher test classification accuracy. Next, we compare the class-wise prediction accuracy of the best stand-alone model (VGG16), and the best GRU, LMU model from Table 1 in Fig. 3. We see that GRU, LMU has more number of classes in higher prediction accuracy bands as compared to VGG16. For the individual class-level classification details, we refer the reader to Supplementary Figure 4.

**Table 2:** Model complexity.

Stand-alone models							
		ResNet18		ResNet50		VGG16	
		14		24		134	
Temporal correlation with CNN/RNN							
Size	TCNN	LSTM		GRU		LMU	
		Layers					
		1	3	1	3	1	3
		CNN1					
S	3.8	1.2	1.4	1.1	1.3	1.0	1.1
L	9.8	2.8	7.0	2.4	5.1	1.6	2.8
		CNN2					
S	5.3	2.9	3.1	2.8	3.0	2.6	2.7
L	11.3	4.8	9.0	4.3	7.5	3.3	4.5
		CNN3					
S	17.6	15.4	15.7	15.3	15.5	15.0	15.1
L	23.6	18.2	22.4	17.4	20.5	15.9	17.0

Total number of trainable parameters (in Millions) for different models.

### Visualizing the learned representations

**Figure 4:**
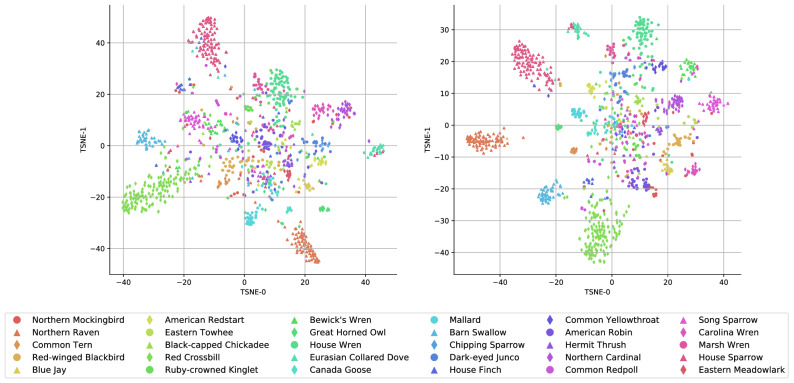
2D projection of learned representations. We visualize samples from our test set using a 2D projection of the representation using a t-SNE plot for 30 bird species with the most number of examples. The embeddings are shown for CNN3+LMU in the *left* plot, and for CNN3+GRU in the *right* with a hidden layer size of 512 for each model.

We now analyze the representations learned by the trained models for different bird species. For each audio sample, we obtain the representation by taking the output of the penultimate layer of the model, and in Fig. 4 we show the t-SNE embeddings in two dimensions for 1522 test samples over 30 bird species. The 30 bird species with the most number of samples are picked from a total of 100 species data. The embedding for two different models CNN3+(LMU, GRU) with a hidden size of 512 is shown in the left and right plots, respectively. For both models, we see that the bird species like Red Crossbill, Northern Raven and House Sparrow that have distinct calls appear in tight-knit clusters (for further bird species related information we refer the reader to *Birds of the World*^26^). On the other hand, species like Northern Mockingbird which belong to the mimic-thrush family, *Mimidae*, have spread-out examples due to the heterogeneity of their calls. We find Northern Mockingbird examples in clusters belonging to several species of Wrens, the Blue Jay and American Robin. Further, House Wren and Marsh Wren examples are projected close together by both methods due to their similar calls, whereas Carolina Wren and Bewick’s Wren are farther. Using the embedding plot we can further identify the clustered species and the species that are close to each other which could provide insights to the bird ecologists. The complete embedding plots with all the species is provided in the supplementary materials.

### Analyzing memory

**Figure 5:**
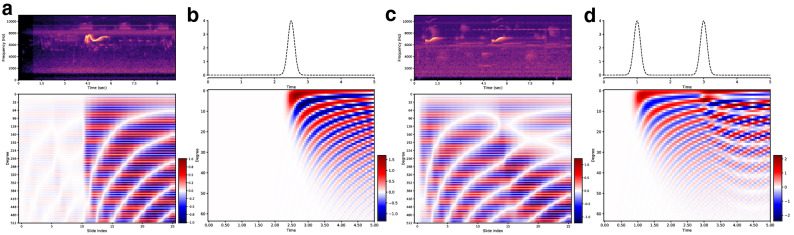
LMU memory channels. LMU memory channels behavior vs time for input signals in the form of pulse. For each subplot, the input is shown in the top and the bottom is memory channels value vs time. A bird sound test sample with single/double spectrogram pulse is shown in (**a**,**c**), respectively. The time in spectrogram is synchronized with the slide index in accordance with the chosen values of $(W_{s},H_{s})$ (see “Methods”). Simulated version of single/double pulse input is shown in (**b**,**d**), respectively.

The deep learning models like the ones we have seen in the previous section deliver good performance. But understanding their mechanism i.e. interpreting what the models have learned, is still difficult. The gating mechanisms employed in LSTM and GRU are difficult to interpret w.r.t how they act upon different input signals like sounds. On the other hand, an RNN like LMU is based on entirely different machinery that employs a state-space model and updates the memory channels using the dynamical Eq. (3) with matrices *A*, *B* in (3) constructed using Legendre polynomials. Another interpretation for the LMU memory mechanism which makes more sense is: the LMU memory Eq. (3) projects the entire input signal history into a fixed number of orthogonal Legendre polynomials^27^ in an online fashion. The projection is made at each time-step, and to avoid the repeated computation of projections, the dynamical equation in (3) is used (see “Methods”). We demonstrate this projection behavior of the LMU in Fig. 5. We see in Fig. 5a that the trained LMU model starts to populate the memory channels upon the first arrival of the pulse in the spectrogram. For the later time points, the memory channel values are transformed to register the signal history. In Fig. 5b, we demonstrate this behavior by simulating a pulse input and projecting the signal history at any time *t* onto 64 orthogonal Legendre polynomials but *without* using the dynamical Eq. (3). Before the t = 2 s time-point, the projections are zero as there is no signal history. We then see the patterns of memory channels (similar to Fig. 5a) as the pulse arrives. A similar behavior is shown for the bird spectrogram with two pulses in Fig. 5c and a simulated version of two pulses in Fig. 5d. We see that the arrival of the second pulse changes the evolution pattern of the memory channels.

**Figure 6:**
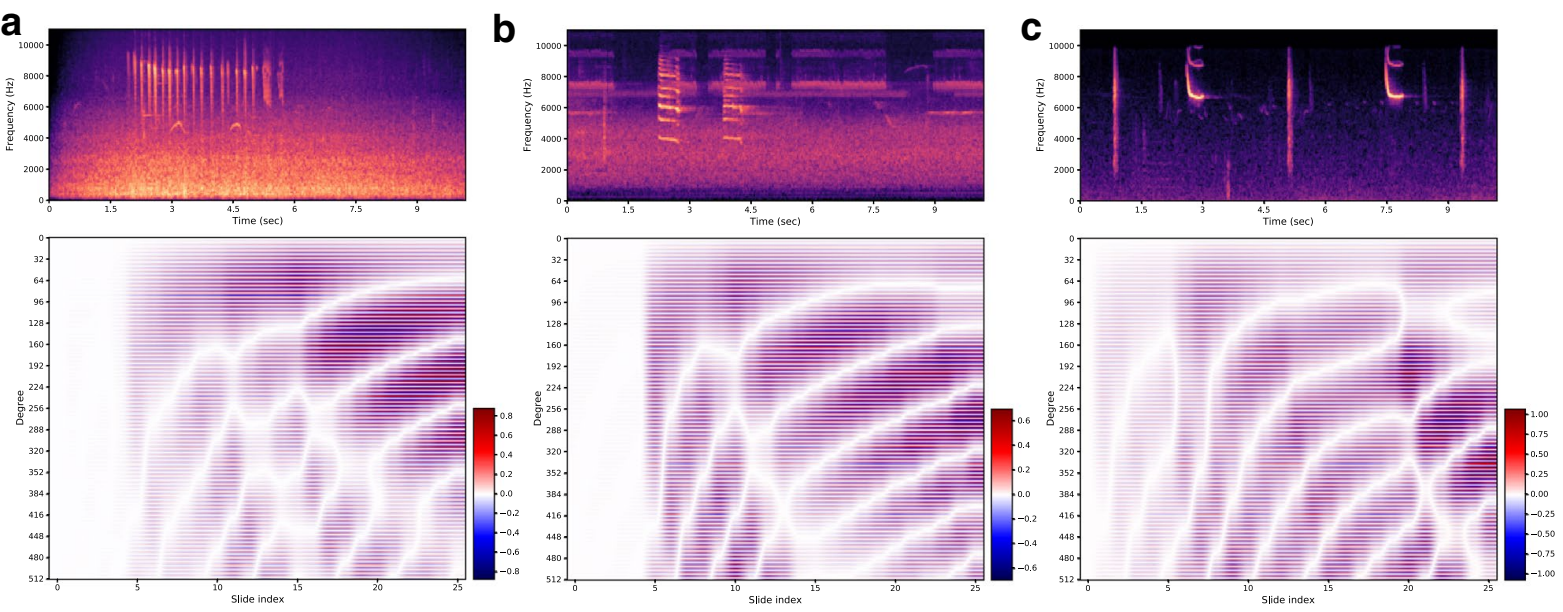
LMU memory channels for real examples. Variations of LMU memory channel values with time for three different bird species spectrograms, Anna’s Hummingbird in (**a**), Blue Jay in (**b**), and Red Winged Blackbird in (**c**). The time in spectrogram is synchronized with the slide index in accordance with the chosen values of $(W_{s},H_{s})$ (see “Methods”).

The LMU memory channel values with time are compared for three different bird species samples in Fig. 6. We see that, irrespective of the different bird species, the memory starts populating when the significant energy in the spectrograms is first detected. Some misalignment exists between the beginning of spectrogram pulses and the corresponding response in the memory channels due to the granularity of the chosen stride parameters $(W_{s}, H_{s})$ (see “Methods” for more details). We make the following two conclusions: (i) for a pulse-like behavior where the spectrogram has energy concentrated in a short-time duration, the memory channels have fading in a smooth fashion as we see in Fig. 6b. While for the spectrograms with energy spread out in time, we see more frequent changes in the memory channels with circular patterns in Fig. 6a. Next, (ii) compared to the double pulse example, as we see in Fig. 5c, where the spectrogram has energy in a narrow frequency range of 6–7 KHz, the case where energy is scattered in a wider range of 4–9 KHz in Fig. 6b and 8–10 KHz in Fig. 6c has different response for the memory channels.

## Methods

We describe the input representation and neural network architectures in detail below. The code from our implementation is available at: https://github.com/microsoft/bird-acoustics-rcnn.

### Spectrograms

The frequency transformation of a time-domain signal using mel-spectrograms has been shown to be better than short time Fourier transform (STFT) and mel-frequency cepstral coefficients (MFCCs)^28^ in prior works^29,30^. We compute mel-spectrograms using librosa^23^ for the 7 s clipped audio signals. The audio is re-sampled at 32KHz and a total of 128 mel filter banks were used. The Fast Fourier Transform (FFT) length is taken to be 2048, and the hop-length for computing the spectrogram is set to 512.

### Models

Each of the models is trained using Adam optimizer with a learning rate of $10^{-4}$ for a total of 50 epochs. The model with the best validation accuracy is chosen for testing.

#### Stand-alone

The ImageNet based models, for example, VGG16, ResNet are used as classifiers with the spectrogram images as the 2-dimensional input. The spectrograms are scaled to $224\times 224$ images with 3 channels for R,G,B. The neurons in the final layer are set to the number of classes in the dataset. Since our processed CBC2020 dataset has 100 classes, the output layer has 100 neurons.

#### Hybrid

**Figure 7:**
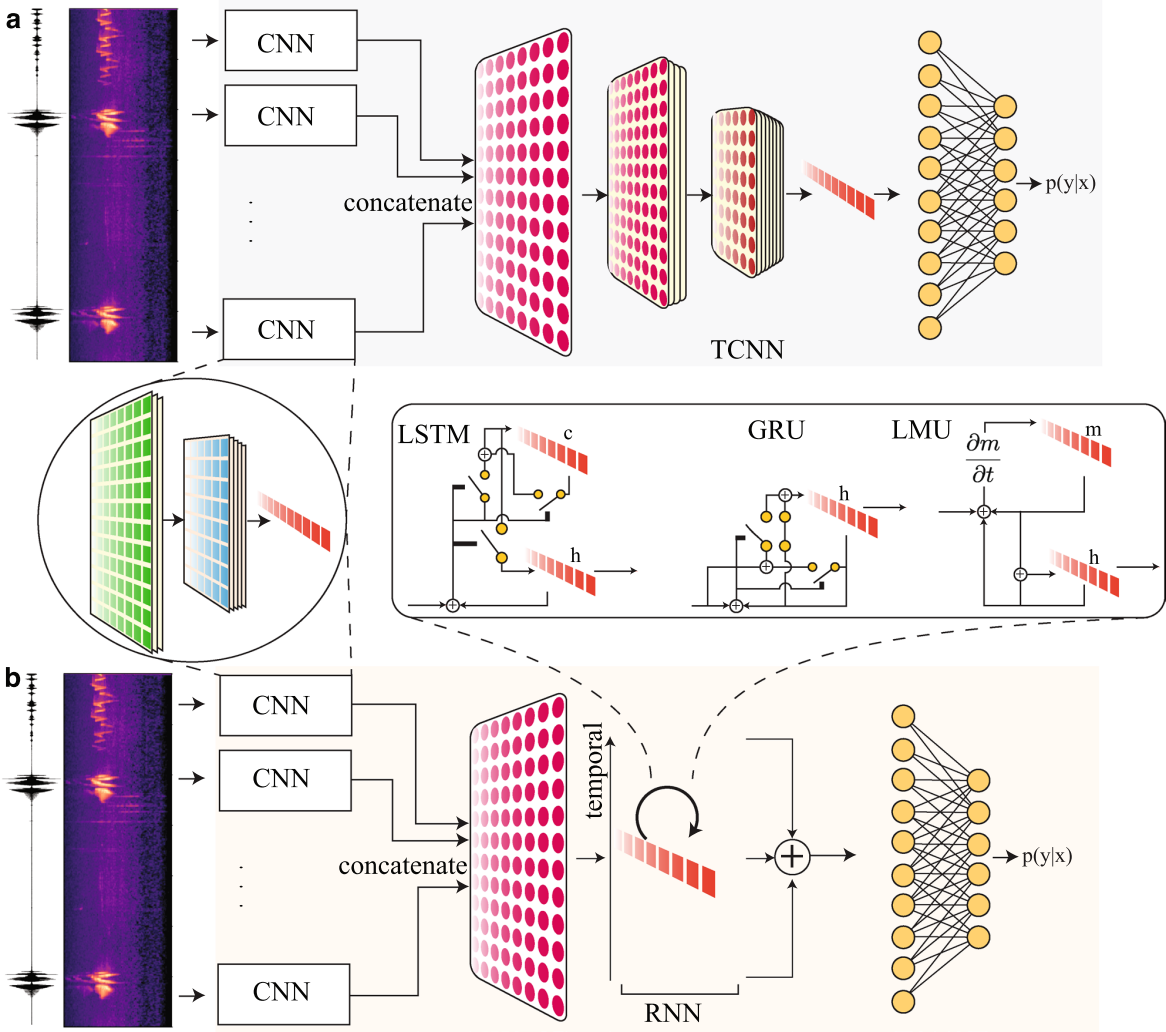
A schematic of hybrid models for classification. Model pipeline using CNN for representation and using another TCNN in (**a**), RNN in (**b**) for temporal correlation extraction. The CNN outputs are concatenated before feeding to the temporal layer in (**a**,**b**).

The hybrid models use a sliding window mechanism for the input due to the temporal component. The raw audio clip is traversed via a sliding window of length $W_s$ and hop length $H_s$. Each hop results in a clipped audio of length $W_s$ which is transformed to the frequency domain using mel-spectrograms. The values of $(W_{s}, H_{s})$ used in this work are (500, 250) ms. For a 7-s audio clip, a total of 26 slides are made with the used values of $W_s, H_s$. Each slide of the spectrogram results in a $128\times 32$ single channel 2-dimensional input. After input, the hybrid models have three parts, (i) Representation, (ii) Temporal correlation, and (iii) Classification. The representation block uses a CNN to generate representative features from the input slides. After concatenating the representative feature vectors from multiple slides, the resulting 2-dimensional array is used as an input to the next Temporal correlation block. The schematic for hybrid models is shown in Fig. 7. The output from the temporal correlation block is fed to the final classification block to produce the softmax outputs.

#### Representation models

In this work, we experiment with three CNN architectures (CNN1, CNN2, and CNN3) of different lengths for the representation block as shown in Table 3. The convolution layer with its corresponding number of filters (X) is shown as ‘Conv-X’. The filter size is $3\times 3$ for each convolution layer in all the three models. Every convolution filter layer is followed by a Batch normalization layer and ReLU operation. The MaxPool is set to downsample with a factor of 2 for each model. Each model ends with an Adaptive Average Pool (AAvgPool) layer with the fixed output configuration of (2, 1).

**Table 3:** Representation models.

CNN1	CNN2	CNN3
Conv-32	Conv-32	Conv-64
	Conv-64	Conv-64
MaxPool	MaxPool	MaxPool
Conv-64	Conv-64	Conv-128
Conv-64	Conv-64	Conv-128
	Conv-64	
MaxPool	MaxPool	MaxPool
Conv-128	Conv-128	Conv-256
Conv-128	Conv-128	Conv-256
Conv-128	Conv-128	Conv-256
MaxPool	MaxPool	MaxPool
Conv-128	Conv-128	Conv-256
Conv-128	Conv-128	Conv-256
Conv-128	Conv-128	Conv-256
AAvgPool	MaxPool	MaxPool
	Conv-256	Conv-512
	Conv-256	Conv-512
	Conv-256	Conv-512
	AAvgPool	AAvgPool

The model structures of the three CNNs used for representation.

#### Temporal models

The temporal block either uses CNN (as shown in Fig. 7a), or RNN (as shown in Fig. 7b). In this work, the models using CNN in the temporal block use one of the three networks: TCNN1, TCNN2, or TCNN3 as shown in Table 4. The convolution layer with its corresponding number of filters (X) is shown as ’Conv-X’. The filter size is $3\times 3$ for each convolution layer in all the three models. Every convolution filter layer is followed by a Batch Normalization layer and ReLU operation. The MaxPool is set to downsample with a factor of 2 for each model.

1 $$\begin{aligned} f_{t}&= \sigma (W_{fx}x_{t} + W_{fh}h_{t-1} + b_{f}),\nonumber \\ i_{t}&= \sigma (W_{ix}x_{t} + W_{ih}h_{t-1} + b_{i}),\nonumber \\ o_{t}&= \sigma (W_{ox}x_{t} + W_{oh}h_{t-1} + b_{o}),\nonumber \\ \tilde{c}_{t}&= \sigma (W_{cx}x_{t} + W_{ch}h_{t-1} + b_{c}),\nonumber \\ c_{t}&= f_{t}\odot c_{t-1} + i_{t}\odot \tilde{c}_{t},\nonumber \\ h_{t}&= o_{t} \odot \text {tanh}(c_{t}). \end{aligned}$$

2 $$\begin{aligned} z_{t}&= \sigma (W_{zx}x_{t} + W_{zh}h_{t-1} + b_{z}),\nonumber \\ r_{t}&= \sigma (W_{rx}x_{t} + W_{rh}h_{t-1} + b_{r}),\nonumber \\ \tilde{h}_{t}&= \text {tanh}(W_{ch}(r_{t}\odot h_{t-1})+ W_{cx}x_{t} + b_{h}),\nonumber \\ h_{t}&= (1-z_{t})\odot h_{t-1} +z_{t}\odot \tilde{h}_{t}. \end{aligned}$$

3 $$\begin{aligned} h_{t}&= \text {tanh}(W_{x}x_{t} + W_{h}h_{t-1} + W_{m}m_{t}),\nonumber \\ u_{t}&= e_{x}^{T}x_{t} + e_{h}^{T}h_{t-1} + e_{m}^{T}m_{t},\nonumber \\ m_{t}&= \bar{A}m_{t-1} + \bar{B}u_{t}. \end{aligned}$$

**Table 4:** Temporal models.

TCNN1	TCNN2	TCNN3
Conv-64	Conv-64	Conv-64
Conv-64	Conv-64	Conv-64
MaxPool	MaxPool	MaxPool
Conv-128	Conv-128	Conv-128
Conv-128	Conv-128	Conv-128
Conv-128	Conv-128	Conv-128
MaxPool	MaxPool	MaxPool
Conv-128	Conv-256	Conv-256
Conv-128	Conv-256	Conv-256
Conv-128	Conv-256	Conv-256
MaxPool	MaxPool	MaxPool
Conv-256	Conv-256	Conv-512
Conv-256	Conv-256	Conv-512
Conv-256	Conv-256	Conv-512

The CNN model structures to learn the temporal component.

The hybrid models with a RNN temporal block use one of three different RNN architectures, namely LSTM, GRU, and LMU. The LSTM uses a hidden state *h* and also maintains a cell state *c*. The recursive update equations for the LSTM are shown in Eq. (1). The GRU has a compact gating mechanism compared to the LSTM and has two gates. The update equations for the GRU are stated in Eq. (2). The LMU uses a memory concept and updates the memory using projections onto Legendre polynomials. The update equations (as shown in Eq. (3)) are less expensive in terms of trainable parameters due to the fixed values of $\bar{A}, \bar{B}$ matrices. We refer the reader to original work^31^ for more details.

Finally, the output of the temporal block is used as an input to the classification block which implements a fully-connected multi-layer perceptron (MLP). The classification block has one layer of 512 neurons with ReLu non-linearity followed by a dropout layer (with probability 0.5) and an output layer of neurons that depends on the number of classes in the dataset. In the case of the temporal block being RNN, the outputs at all time-steps are summed before feeding to the classification block.

### Analyzing memory

An alternate way to interpret the LMU mechanism, apart from the state-space representation, is projecting the memory onto a fixed set of orthogonal basis. Hence, the LMU works by repeated projection of the entire history of hidden states $h_{t}$ and the input $x_{t}$, $t\ge 0$ onto a fixed number of Legendre polynomials. The Legendre polynomials are a class of orthogonal polynomials with the following property.

4 $$\begin{aligned} \int \limits _{-1}^{1}P_{m}(x)P_{n}(x)dx ={\left\{ \begin{array}{ll} 0, &{} m\ne n\\ \frac{2}{2n+1}, &{} m=n \end{array}\right. }, \end{aligned}$$

where $P_{m}(x)$ is the Legendre polynomial with degree *m*. The Legendre polynomials also satisfy the following

5 $$\begin{aligned} P^{\prime }_{m+1}&= (m+1)P_{m} + xP^{\prime }_{m}, \end{aligned}$$

6 $$\begin{aligned} (2m+1)P_{m}&= P^{\prime }_{m+1} - P^{\prime }_{m-1}, \end{aligned}$$

7 $$\begin{aligned} P_m(1)&= 1,\quad P_{m}(-1) = (-1)^m. \end{aligned}$$

For a signal *f*(*t*), its projection along the *m*th degree Legendre polynomial is defined as

8 $$\begin{aligned} c_{m}(t) = \int \limits _{0}^{t}f(x)P_{m}(x)dx. \end{aligned}$$

In Fig. 5b,c we use Eq. (8) to show the projection coefficient variations over time with the maximum degree of 64. Directly evaluating the projections at each time-step *t* using Eq. (8) is not computationally feasible, especially when the time-horizon is large. However, due to the recurrence properties of the Legendre polynomials as shown in Eqs. (5) and (6) a dynamical equation similar to Eq. (3) can be constructed to update the projection coefficients recursively.

## Related work

During the past decade, deep convolutional neural network (CNN) architectures have demonstrated great potential in classification problems as well as other tasks, such as object detection and image segmentation. Some well-known CNN architectures include VGG16^24^, ResNet^25^, and DenseNet^32^, among others. These models can successfully extract complex features from images and differentiate a high number of potentially similar classes, and have recently gathered popularity in the field of bioacoustics as well. For example, there are some works using CNN, either based on the well-known architectures or customized architectures, to detect and classify the presence of whale acoustics^17,33^, or classify calls from different bird species^20,34,35^.

While CNN models usually include millions of parameters, training such a model typically requires a sufficiently large amount of data in order to achieve good performance. However, it is a time-consuming and expensive endeavor to obtain a manually labeled dataset in bioacoustics, and it may also be very challenging to collect enough labeled data in practice, especially if a species rarely calls or if a species is rare. Given this scenario, some bioacoustics research works used other techniques in addition to CNN, including transfer learning with fine-tuning^36–39^, pseudo-labeling^40^, and using few-shot learning approaches^41^.

Existing literature in recurrent and convolutional neural networks has extensively explored the classification task on the sequence and time-series datasets. While not explicitly modeling the temporal dependencies, fully convolutional networks, and ResNet architectures are shown to perform well for time-series classification^42^. Vanilla recurrent neural nets were designed to capture temporal dependencies for sequence data^43,44^. However, they suffer from vanishing/exploding gradients^45^. As a remedy, more sophisticated recurrent neural net units that implement a gating mechanism, such as a long short-term memory (LSTM) unit^46^ and gated recurrent unit (GRU)^47^ are proposed in the literature. For the audio classification task, a gated Residual Networks model that integrates ResNet with a gate mechanism was shown to be promising^48^. To efficiently handle the temporal dependencies, the Legendre Memory Unit (LMU) was proposed as a novel memory cell for recurrent neural networks with theoretical guarantees for learning long-range dependencies^31,49^. It dynamically maintains information across long windows of time using relatively few resources via orthogonalizing its continuous-time history.

Hybrid models leverage the strengths of both convolutional and recurrent neural networks for learning from temporal or sequence data. They use convolutional layers to extract local patterns at each time-point and then couple the learned representations over multiple time-points using a recurrent component. As compared to the models that use another CNN layer to aggregate the representations across time-steps, the use of a recurrent structure allows them to better capture long-term dependencies in the input. Various choices of recurrent components have been tried, such as LSTMs, GRUs. Some of these are: a one-dimensional CNN coupled with a GRU^50^, an LSTM coupled to a CNN for audio classification^51^, a recurrent structure that is based on GRUs, with temporal skip connections to extend the temporal span of the information flow for modeling multi-dimensional time-series^52^. A variety of CNN and RNN models are explored in^53^ where superior performance of deep nets compared to some traditional machine learning models is demonstrated for automatic detection of endangered mammals species based on spectrograms. Hybrid models have shown improvements in accuracy over the baseline CNN-only models on various sound detection tasks in the recent literature^54,55^. Further, for the task of music tagging, Choi et al.^56^ show that their convolutional recurrent neural network (CRNN), that also involves a GRU, does better in terms of training time and the number of parameters compared to the purely CNN-based prior architectures. Specifically for bird sounds, some recent works^57,58^ have explored the approach of CRNNs for detecting the presence/absence of a bird call in the audio clip, usually termed as Bird Audio Detection (BAD). The methods of BAD can be used as a preliminary step towards building models for species-level classification.

## Conclusion

We present a comprehensive study of hybrid deep learning models on a large bird acoustics dataset Cornell Bird Challenge (CBC)2020. Deep learning models offer high predictive capability and at the same time leads to a design with a more automated pipeline. Although Imagenet-based models have been successfully applied for sound classification through spectrograms, they work on individual images and do not capture the temporal dependencies across time-points. We found that for bird acoustics data (CBC2020), hybrid models with an explicit temporal layer perform better. The hybrid models, when compared to the Imagenet-based models, offer a two-fold advantage of reduced model size as well as higher test accuracy. This leads us to conclude that larger models do not always result in a better test accuracy. In the context of RNNs, in most cases, one or two layers were sufficient and resulted in more accurate models. In addition to the gating mechanisms based RNNs like Long-Short term memory (LSTM), and Gated recurrent units (GRU), we also present a novel hybrid model utilizing Legendre memory units (LMU). The LMU works on a different mechanism of orthogonalizing memory and offers the further advantage of long-range dependence as well as reduced model parameters. We have presented an empirical analysis of how LMU memory channels behave with time for different spectrogram inputs.

We have also analyzed how models are representing different bird species sound samples through the embedding plot. We found out that the birds with distinct calls (for example, Red crossbill, Northern raven, etc.) are packed together and are distant from each other. Some bird species with assorted calls are spread across other species representations.

The hybrid models with a built-in temporal layer have an additional requirement of a longer time sequence. For shorter time-series, learning dependencies across time components was found out to be difficult through RNNs. We also found that adding the attention mechanisms to the hybrid models with RNN does not help with the CBC2020 dataset. Part of the reason could be that the bird call location in the input audio is very uncertain, even in the clipped version. In future work, we would extend the current models to detect multiple species of bird calls and also apply the same analysis to different sound datasets, for example, marine animals detection.

## Supplementary information

Supplementary Information.
