# Supplementary material for: Comparing recurrent convolutional neural networks for large scale bird species classification
Source: Sci Rep. 2021 Aug 24;11:17085. doi: 10.1038/s41598-021-96446-w (PMC8385065; doi:10.1038/s41598-021-96446-w)
Supplement: Supplementary file 1 — Supplementary Information. [file 41598_2021_96446_MOESM1_ESM.pdf]

Supplementary Materials: Comparing Recurrent Convolutional Neural Networks for large scale Bird species classification

Gaurav Gupta[1,*], Meghana Kshirsagar[2], Ming Zhong[2], Shahrzad Gholami[2], and Juan Lavista Ferres[2]

[1]Ming Hsieh Department of Electrical and Computer Engineering, University of Southern California, Los Angeles, CA 90089, USA.
[2]AI for Good Research Lab, Microsoft, Redmond, WA 98052, USA.
[*]ggaurav@usc.edu

Supplementary Materials

stand-alone models		
ResNet18	ResNet50	VGG16
0.516	0.537	0.619

Temporal correlation with RNN												
	LSTM			GRU			LMU			GRU+LMU		
Size	Layers											
	1	2	3	1	2	3	1	2	3	1	2	3
	CNN1											
128	0.573	0.563	0.555	0.600	0.580	0.580	0.566	0.548	0.541	–	0.573	0.561
256	0.603	0.602	0.589	0.618	0.612	0.603	0.591	0.581	0.576	–	0.615	0.606
512	0.621	0.615	0.608	0.634	0.644	0.630	0.607	0.610	0.594	–	0.635	0.630
	CNN2											
128	0.603	0.579	0.551	0.624	0.601	0.598	0.583	0.576	0.557	–	0.586	0.586
256	0.621	0.601	0.573	0.635	0.611	0.626	0.597	0.602	0.590	–	0.622	0.613
512	0.625	0.613	0.602	0.635	0.634	0.638	0.629	0.622	0.602	–	0.633	0.629
	CNN3											
128	0.657	0.619	0.584	0.644	0.639	0.620	0.651	0.638	0.635	–	0.628	0.622
256	0.658	0.630	0.591	0.654	0.654	0.644	0.641	0.639	0.630	–	0.653	0.633
512	0.663	0.635	0.642	0.657	**0.667**	0.654	0.652	0.647	0.643	–	**0.665**	0.650

Table 1. Test accuracy comparison on the CBC dataset: **Top**: Models without any explicit temporal layer. The input is a single spectrogram for sound sample. **Bottom**: A comprehensive comparison of models test accuracy using RNN for temporal correlation. The complexity of CNN used for representation increase from top to bottom. The best accuracy achieved is shown in bold.

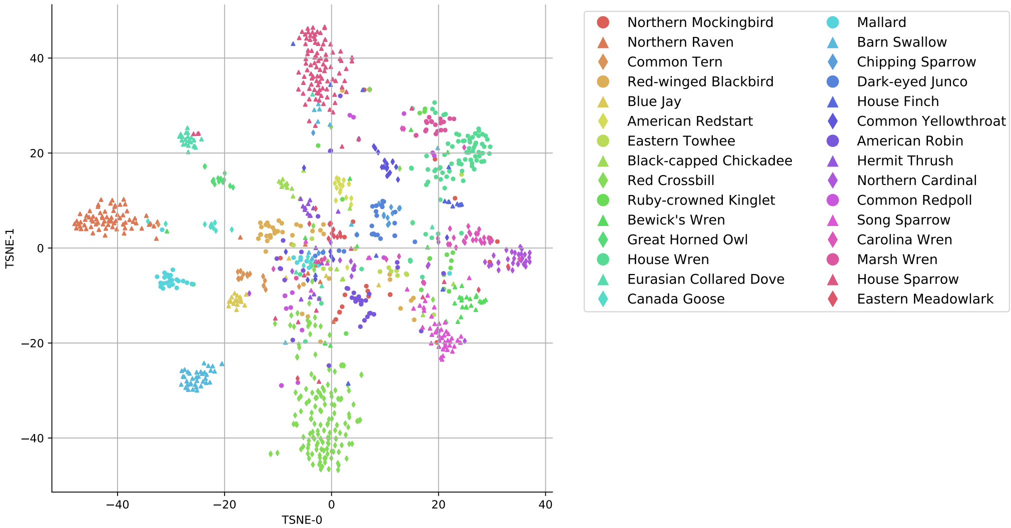

Figure 2. Samples model representation: t-SNE plot along two dimensions for 30 bird species with most number of samples. The test samples embedding is shown for CNN3+LSTM with hidden size of 512.

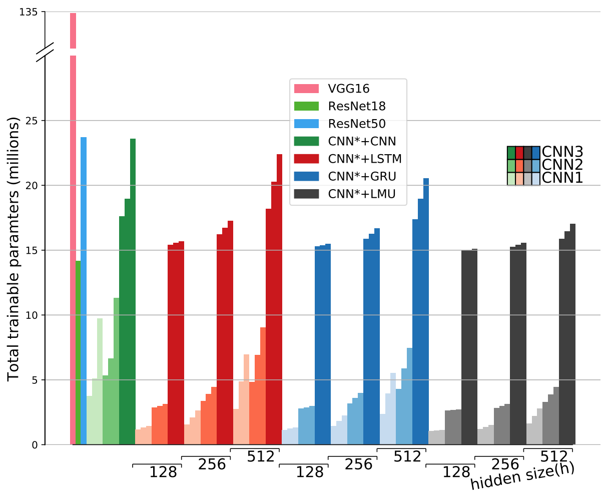

Figure 1. Comparison of models. (b) The model complexity in terms of total trainable parameters.

Temporal correlation with CNN			
Representation	Temporal		
	TCNN1	TCNN2	TCNN3
CNN1	0.489	0.493	0.503
CNN2	0.557	0.544	0.550
CNN3	0.579	0.577	0.614

Table 2. Test accuracy comparison: A comparison of the test accuracy for the CBC dataset using (CNN+CNN) hybrid models of different sizes. From top to bottom the complexity of the CNN layers extracting the representation increases, while from left to right the complexity of the TCNN layer that incorporates temporal correlation increases.

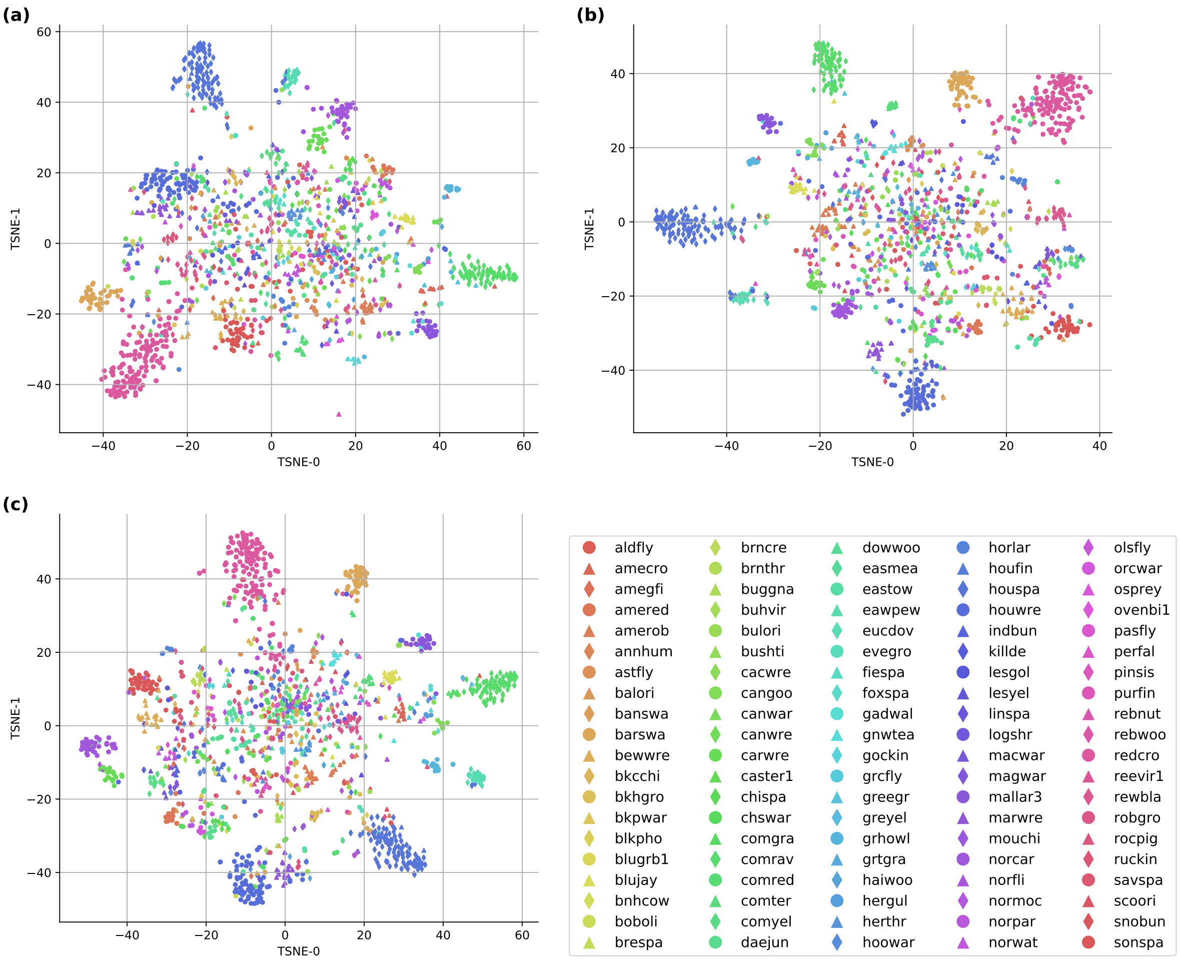

Figure 3. **Samples model representation:** t-SNE plot along two dimensions for 100 bird species. The test samples embedding is shown for CNN3+LMU, GRU, LSTM in (a), (b), (c), respectively, with hidden size of 512. For birds code see Supplementary Table 3, and for further related information we refer[1].

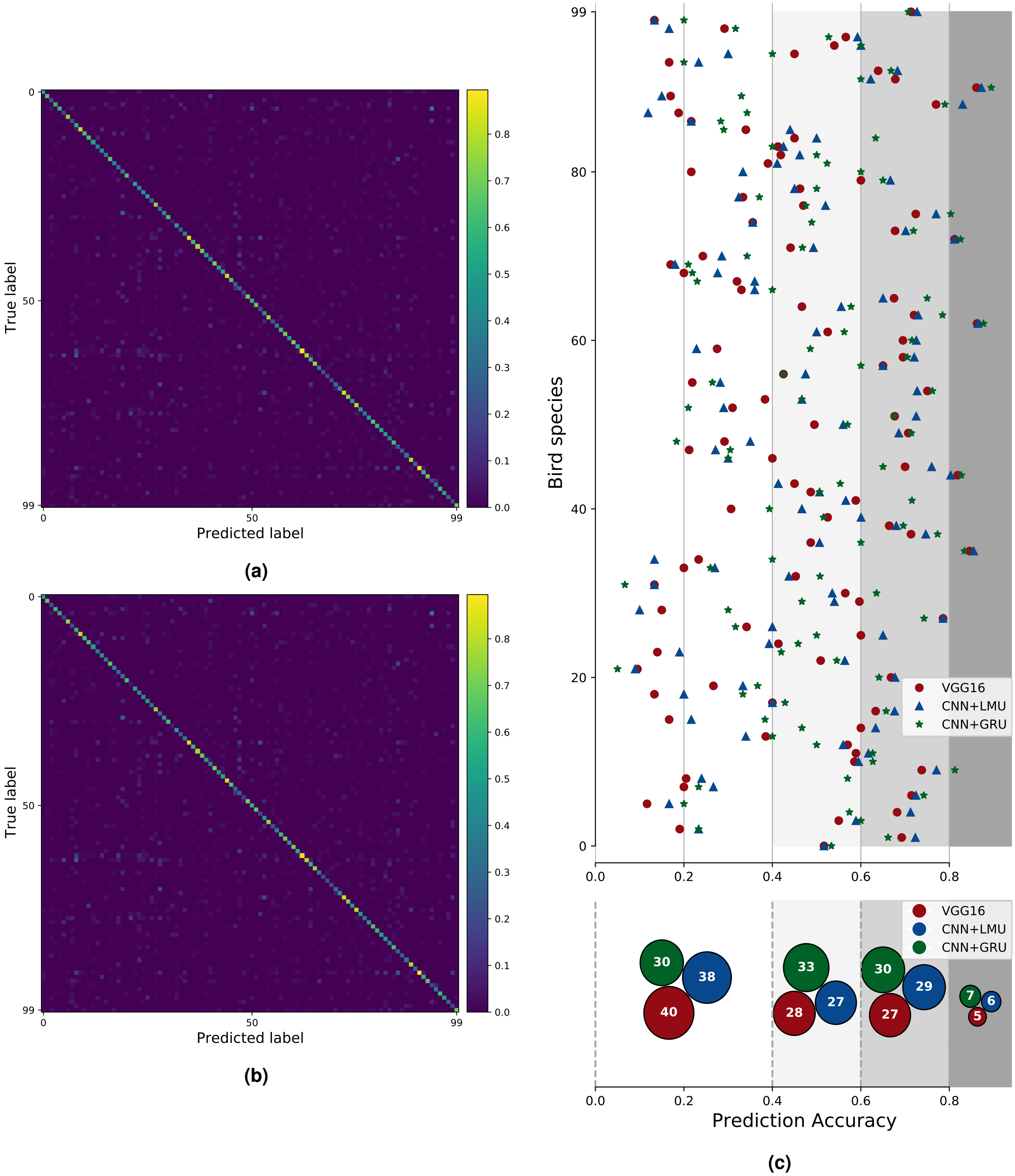

Figure 4. Class-wise prediction accuracy: Confusion matrix for the model predictions over 100 bird species. The values are shown for the best model, according to Table 1, of GRU, LMU in (a), (b), respectively. Class-wise prediction accuracy for the best GRU, LMU, and VGG16 model in (c). Top, bird species code on y-axis vs prediction accuracy on x-axis. In bottom, we show for each model, the percentage of classes that have prediction accuracy in the given shaded brackets. For birds code see Supplementary Table 3, and for further related information we refer[1]. For numerical values of the prediction accuracy, we refer to Supplementary Table 4.

Index	bird code	bird species name
0	aldfly	Alder Flycatcher
1	amecro	American Crow
2	amegfi	American Goldfinch
3	amered	American Redstart
4	amerob	American Robin
5	annhum	Anna's Hummingbird
6	astfly	Ash-throated Flycatcher
7	balori	Baltimore Oriole
8	banswa	Sand Martin
9	barswa	Barn Swallow
10	bewwre	Bewick's Wren
11	bkcchi	Black-capped Chickadee
12	bkhgro	Black-headed Grosbeak
13	bkpwar	Blackpoll Warbler
14	blkpho	Black Phoebe
15	blugrb1	Blue Grosbeak
16	blujay	Blue Jay
17	bnhcow	Brown-headed Cowbird
18	boboli	Bobolink
19	brespa	Brewer's Sparrow
20	brncre	Brown Creeper
21	brnthr	Brown Thrasher
22	buggna	Blue-grey Gnatcatcher
23	buhvir	Blue-headed Vireo
24	bulori	Bullock's Oriole
25	bushti	American Bushtit
26	cacwre	Cactus Wren
27	cangoo	Canada Goose
28	canwar	Canada Warbler
29	canwre	Canyon Wren
30	carwre	Carolina Wren
31	caster1	Caspian Tern
32	chispa	Chipping Sparrow
33	chswar	Chestnut-sided Warbler
34	comgra	Common Grackle
35	comrav	Northern Raven
36	comred	Common Redpoll
37	comter	Common Tern
38	comyel	Common Yellowthroat
39	daejun	Dark-eyed Junco
40	dowwoo	Downy Woodpecker
41	easmea	Eastern Meadowlark
42	eastow	Eastern Towhee
43	eawpew	Eastern Wood Pewee
44	eucdov	Eurasian Collared Dove
45	evegro	Evening Grosbeak
46	fiespa	Field Sparrow
47	foxspa	Red Fox Sparrow
48	gadwal	Gadwall
49	gnwtea	Eurasian Teal

Index	bird code	bird species name
50	gockin	Golden-crowned Kinglet
51	grcfly	Great Crested Flycatcher
52	greegr	Great Egret
53	greyel	Greater Yellowlegs
54	grhowl	Great Horned Owl
55	grtgra	Great-tailed Grackle
56	haiwoo	Hairy Woodpecker
57	hergul	European Herring Gull
58	herthr	Hermit Thrush
59	hoowar	Hooded Warbler
60	horlar	Horned Lark
61	houfin	House Finch
62	houspa	House Sparrow
63	houwre	House Wren
64	indbun	Indigo Bunting
65	killde	Killdeer
66	lesgol	Lesser Goldfinch
67	lesyel	Lesser Yellowlegs
68	linspa	Lincoln's Sparrow
69	logshr	Loggerhead Shrike
70	macwar	MacGillivray's Warbler
71	magwar	Magnolia Warbler
72	mallar3	Mallard
73	marwre	Marsh Wren
74	mouchi	Mountain Chickadee
75	norcar	Northern Cardinal
76	norfli	Northern Flicker
77	normoc	Northern Mockingbird
78	norpar	Northern Parula
79	norwat	Northern Waterthrush
80	olsfly	Olive-sided Flycatcher
81	orcwar	Orange-crowned Warbler
82	osprey	Western Osprey
83	ovenbi1	Ovenbird
84	pasfly	Pacific-slope Flycatcher
85	perfal	Peregrine Falcon
86	pinsis	Pine Siskin
87	purfin	Purple Finch
88	rebnut	Red-breasted Nuthatch
89	rebwoo	Red-bellied Woodpecker
90	redcro	Red Crossbill
91	reevir1	Red-eyed Vireo
92	rewbla	Red-winged Blackbird
93	robgro	Rose-breasted Grosbeak
94	rocpig	Rock Dove
95	ruckin	Ruby-crowned Kinglet
96	savspa	Savannah Sparrow
97	scoori	Scott's Oriole
98	snobun	Snow Bunting
99	sonspa	Song Sparrow

Table 3. Bird species code: The index and bird code mapping for various bird species. For further information we refer *Birds of the World*[1].

Index	bird code	Prediction Accuracy		
		VGG16	GRU	LMU
0	aldfly	0.517	0.533	0.517
1	amecro	0.692	0.662	0.723
2	amegfi	0.190	0.233	0.233
3	amered	0.550	0.600	0.589
4	amerob	0.682	0.574	0.712
5	annhum	0.117	0.200	0.167
6	astfly	0.714	0.742	0.724
7	balori	0.200	0.233	0.267
8	banswa	0.205	0.570	0.240
9	barswa	0.738	0.812	0.771
10	bewwre	0.585	0.627	0.595
11	bkcchi	0.589	0.627	0.617
12	bkhgro	0.570	0.500	0.560
13	bkpwar	0.385	0.400	0.340
14	blkpho	0.600	0.467	0.633
15	blugrb1	0.167	0.383	0.217
16	blujay	0.633	0.657	0.676
17	bnhcow	0.400	0.429	0.400
18	boboli	0.133	0.333	0.200
19	brespa	0.267	0.367	0.333
20	brncre	0.668	0.641	0.677
21	brnthr	0.095	0.050	0.090
22	buggna	0.509	0.545	0.564
23	buhvir	0.140	0.420	0.190
24	bulori	0.414	0.458	0.393
25	bushti	0.600	0.500	0.650
26	cacwre	0.342	0.317	0.400
27	cangoo	0.786	0.743	0.786
28	canwar	0.150	0.300	0.100
29	canwre	0.597	0.467	0.540
30	carwre	0.565	0.635	0.535
31	caster1	0.133	0.067	0.133
32	chispa	0.453	0.508	0.437
33	chswar	0.200	0.260	0.270
34	comgra	0.233	0.400	0.133
35	comrav	0.846	0.834	0.855
36	comred	0.487	0.600	0.507
37	comter	0.713	0.773	0.747
38	comyel	0.664	0.696	0.680
39	daejun	0.525	0.516	0.600
40	dowwoo	0.307	0.393	0.467
41	easmea	0.588	0.715	0.566
42	eastow	0.487	0.507	0.507
43	eawpew	0.450	0.553	0.413
44	eucdov	0.819	0.827	0.803
45	evegro	0.700	0.650	0.760
46	fiespa	0.400	0.300	0.300
47	foxspa	0.212	0.305	0.271
48	gadwal	0.292	0.183	0.350
49	gnwtea	0.707	0.714	0.686
50	gockin	0.495	0.570	0.560
51	grcfly	0.676	0.675	0.725
52	greegr	0.310	0.210	0.290
53	greyel	0.383	0.467	0.467
54	grhowl	0.750	0.762	0.727
55	grtgra	0.219	0.264	0.282
56	haiwoo	0.425	0.425	0.475
57	hergul	0.650	0.600	0.650
58	herthr	0.695	0.705	0.721
59	hoowar	0.275	0.486	0.229
60	horlar	0.695	0.715	0.725
61	houfin	0.525	0.562	0.500
62	houspa	0.863	0.877	0.864
63	houwre	0.720	0.784	0.730
64	indbun	0.467	0.578	0.556
65	killde	0.675	0.750	0.650
66	lesgol	0.330	0.400	0.360
67	lesyel	0.320	0.230	0.360
68	linspa	0.200	0.219	0.276
69	logshr	0.170	0.210	0.180
70	macwar	0.243	0.343	0.286
71	magwar	0.441	0.468	0.493
72	mallar3	0.812	0.825	0.812
73	marwre	0.677	0.719	0.701
74	mouchi	0.356	0.489	0.356
75	norcar	0.724	0.803	0.770
76	norfli	0.470	0.476	0.520
77	normoc	0.334	0.371	0.324
78	norpar	0.463	0.500	0.450
79	norwat	0.600	0.650	0.667
80	olsfly	0.217	0.600	0.333
81	orcwar	0.390	0.524	0.411
82	osprey	0.419	0.500	0.462
83	ovenbi1	0.412	0.400	0.425
84	pasfly	0.450	0.633	0.500
85	perfal	0.340	0.290	0.440
86	pinsis	0.217	0.283	0.217
87	purfin	0.188	0.343	0.119
88	rebnut	0.770	0.790	0.830
89	rebwoo	0.170	0.330	0.150
90	redcro	0.861	0.895	0.872
91	reevir1	0.678	0.600	0.622
92	rewbla	0.639	0.668	0.683
93	robgro	0.167	0.200	0.233
94	rocpig	0.450	0.400	0.300
95	ruckin	0.540	0.600	0.600
96	savspa	0.566	0.527	0.592
97	scoori	0.292	0.317	0.167
98	snobun	0.133	0.200	0.133
99	sonspa	0.713	0.708	0.727

Table 4. Bird species code: The index and bird code mapping for various bird species. For further information we refer *Birds of the World*[1].

References

1. Billerman, S. M., Keeney, B. K., Rodewald, P. G. & Schulenberg, T. S. (eds.) *Birds of the World* (Cornell Laboratory of Ornithology, Ithaca, NY, USA, 2020). https://birdsoftheworld.org/bow/home.
